# MiR-181b-5p Downregulates NOVA1 to Suppress Proliferation, Migration and Invasion and Promote Apoptosis in Astrocytoma

**DOI:** 10.1371/journal.pone.0109124

**Published:** 2014-10-09

**Authors:** Feng Zhi, Qiang Wang, Danni Deng, Naiyuan Shao, Rong Wang, Lian Xue, Suinuan Wang, Xiwei Xia, Yilin Yang

**Affiliations:** 1 Modern Medical Research Center, Third Affiliated Hospital of Soochow University, Changzhou, Jiangsu, China; 2 Department of Neurosurgery, Third Affiliated Hospital of Soochow University, Changzhou, Jiangsu, China; Baylor College of Medicine, United States of America

## Abstract

MicroRNAs (miRNAs) are small, short noncoding RNAs that modulate the expression of numerous genes by targeting their mRNA. Numerous abnormal miRNA expression patterns are observed in various human malignancies, and certain miRNAs can act as oncogenes or tumor suppressors. Astrocytoma, the most common neuroepithelial cancer, represents the majority of malignant brain tumors in humans. In our previous studies, we found that the downregulation of miR-181b-5p in astrocytomas is associated with a poor prognosis. The aim of the present study was to investigate the functional role of miR-181b-5p and its possible target genes. miR-181b-5p was significantly downregulated in astrocytoma specimens, and the reduced expression of miR-181b-5p was inversely correlated with the clinical stage. The ectopic expression of miR-181b-5p inhibited proliferation, migration and invasion and induced apoptosis in astrocytoma cancer cells *in vitro*. The NOVA1 (neuro-oncological ventral antigen 1) gene was further identified as a novel direct target of miR-181b-5p. Specifically, miR-181b-5p bound directly to the 3'-untranslated region (UTR) of NOVA1 and suppressed its expression. In clinical specimens, NOVA1 was overexpressed, and its protein levels were inversely correlated with miR-181b-5p expression. Furthermore, the changing level of NOVA1 was significantly associated with a poor survival outcome. Similar to restoring miR-181b-5p expression, downregulating NOVA1 inhibited cell growth, migration and invasion. Overexpression of NOVA1 reversed the inhibitory effects of miR-181b-5p. Our results indicate that miR-181b-5p is a tumor suppressor in astrocytoma that inhibits tumor progression by targeting NOVA1. These findings suggest that miR-181b-5p may serve as a novel therapeutic target for astrocytoma.

## Introduction

Astrocytomas are the most common primary brain tumors in the central nervous system [1]. The prognosis of patients with astrocytoma is closely related to the WHO tumor grade, and patients with glioblastoma, the most common histological type, have a poor prognosis, with a median survival of approximately 15 months [2]. Thus, efforts to better understand the biological basis of astrocytoma progression may provide important clinically relevant insights into disease management.

MicroRNAs (miRNAs) are a class of highly conserved, single-stranded, small, noncoding RNA molecules known as endogenous regulators of post-transcriptional gene expression that regulate expression through translational repression and messenger RNA cleavage [3]–[4]. It has been widely accepted that miRNAs play pivotal roles in various biological processes, including development, cell proliferation, differentiation, apoptosis and metabolism [4]. Accumulating evidence also suggests that miRNAs participate in the tumor angiogenesis, invasion and metastasis of human malignancies, acting as oncogenes or tumor suppressors depending on their targets, information that may provide insight into the diagnosis and prognosis of human cancers [5]–[6]. Our previous studies indicated that miR-181b-5p is downregulated in astrocytoma and that this reduced expression of miR-181b-5p is associated with a poor survival outcome, suggesting that miR-181b-5p may act as a tumor suppressor during astrocytoma development and/or progression [7]. miR-181b-5p belongs to the miR-181 family, which includes miR-181a-5p, miR-181b-5p and miR-181c-5p. However, whether miR-181b-5p is a tumor suppressive or oncogenic miRNA remains controversial, and the regulatory mechanism underlying miR-181b-5p-mediated function remains to be elucidated in different cancers. Recently, miR-181b-5p was found to inhibit glioma cell proliferation, migration, invasion and tumorigenesis by targeting *IGF-1R* [8] and to reduce chemoresistance to temozolomide in glioma cells by targeting *MEK1* [9]. As it is well known that miRNAs regulate the expression of multiple target genes and affect a variety of cellular pathways, further research is thus required to fully understand their contributions to this malignancy.

In this study, we clearly demonstrate that miR-181b-5p functions as a tumor suppressor miRNA in astrocytoma. The tumor-suppressive effect was mediated via the repression of NOVA1 (neuro-oncological ventral antigen 1). Our findings provide valuable clues toward understanding the mechanisms of astrocytoma pathogenesis and present an opportunity for the development of new effective clinical therapies.

## Materials and Methods

### Human tissue samples

Surgically excised tumor specimens from 90 patients with astrocytomas and normal adjacent tissues (NATs) from 25 astrocytoma patients were collected in the Department of Neurosurgery of the Third Affiliated Hospital of Soochow University, China. The cases included 8 patients with grade I, 26 with grade II, 33 with grade III and 23 with grade IV astrocytomas. The histological grading was performed on the basis of World Health Organization (WHO) criteria. The collected tissues were immediately snap-frozen in liquid nitrogen and stored at -80°C. Written informed consent was obtained from all of the patients or their representatives before the study, which was approved by the Research Ethics Board of the Third Affiliated Hospital of Soochow University.

### Cell culture

The human astrocytoma cell lines U251 and U87 were purchased from Cell Resource Centre of the Shanghai Institutes for Biological Sciences of the Chinese Academy of Sciences. All of the cells were cultured in Dulbecco's Modified Eagle's Medium (Invitrogen, USA) supplemented with 10% fetal bovine serum (Hyclone, USA) in a humidified 37°C incubator that was maintained at 5% CO_2_.

### Oligonucleotides and cell transfection

The oligonucleotide miR-181b-5p mimic (pre-miR-181b-5p), mimic negative control (pre-ncRNA), miR-181b-5p inhibitor (anti-miR-181b-5p) and inhibitor negative control (anti-ncRNA) were purchased from GenePharma (Shanghai, China). A small interfering RNA (siRNA) targeting NOVA1 (si-NOVA1) and a NOVA1 vector encoding the entire coding sequence without the 3′-UTR were designed and synthesized by Invitrogen (Invitrogen, USA). A scrambled siRNA (si-NC) and a control vector were included as negative controls. miRNAs, siRNAs and vectors were transfected into cells at the indicated concentrations using Lipofectamine 2000 (Invitrogen, Carlsbad, CA, USA) according to the manufacturer's instructions. Total RNA was isolated at 24 h post-transfection. Total cellular protein was isolated at 48 h after transfection.

### RNA isolation and quantitative real-time PCR

Total RNA was extracted from cultured cells or clinical samples using TRIzol Reagent (Invitrogen) according to the manufacturer's protocols. The RNA molecules were then treated with RNase-free DNase (TaKaRa, Dalian, China) using a standardized protocol. The levels of mature miR-181b-5p were quantified using Taqman miRNA probes (Applied Biosystems), as previously reported [10]. The U6 small nuclear RNA was selected as a control. For the analysis of NOVA1 and β-actin, quantitative real-time PCR (qRT-PCR) was performed using an Applied Biosystems 7500 Sequence Detection System (Applied Biosystems, Foster City, CA, USA) with SYBR green dye (Invitrogen). The sequences of the sense and antisense primers used for the amplification of NOVA1 and β-actin were as follows: NOVA1 (sense), 5'- TACTGAGCGAGTGTGCTTGAT-3', NOVA1 (antisense), 5'- GTCTGGGGTTGTAGAATGCTG-3'; β-actin (sense), 5'-AGGGAAATCGTGCGTGAC-3', and β-actin (antisense), 5'-CGCTCATTGCCGATAGTG-3'. All of the reactions were performed in triplicate.

### miR-181b-5p target prediction

The analysis of predicted microRNA targets was determined using algorithms from TargetScan [11], PicTar [12] and microRNA.org [13].

### Western blotting

Total cellular protein was isolated using RIPA lysis buffer (Sigma-Aldrich Inc., St Louis, MO) at 48 h after transfection. Western blotting analyses were performed using conventional protocols, as previously described [14]. Briefly, a rabbit monoclonal β-actin antibody (Cell Signaling Technology, USA), rabbit polyclonal NOVA1 antibody (Abcam, UK) and secondary rabbit IgG-HRP (Sigma, USA) were applied at 1∶1,000, 1∶500 and 1∶5,000 dilutions, respectively. The Quantity One analysis program (Bio-Rad, USA) was used to obtain the quantitative data.

### Luciferase assay

The entire human NOVA1 3′-untranslated region (3′-UTR) was amplified by PCR using human genomic DNA as the template. The PCR products were inserted into the p-MIR-report plasmid (Ambion). Efficient insertion was confirmed by sequencing. For luciferase reporter assays, cells were cultured in 6-well plates, and each well was transfected with 2 µg of firefly luciferase reporter plasmid, 2 µg of β-galactosidase expression vector (Ambion) and equal amounts of pre-ncRNA, pre-miR-181b-5p, anti-ncRNA or anti-miR-181b-5p using Lipofectamine 2000 (Invitrogen). The β-galactosidase vector was used as a transfection control. At 24 h post-transfection, the cells were assayed using luciferase assay kits (Promega, Madison, WI, USA). The data depicted represent three independent experiments performed on different days.

### Cell viability assay

Cells in the logarithmic phase of growth were seeded at 2000 per well in 96-well plates and cultured for 12, 24, 48 and 72 h. Next, 20 µl MTT (5 mg/mL) was added to each test well and incubated for 4 h at 37°C. The supernatant was discarded, and 150 µl of dimethyl sulfoxide (DMSO) was added to each well to solubilize the crystals for 10 min at room temperature. The optical density (OD) was measured at a wavelength of 570 nm.

### Wound-healing assay

Cells were cultured to 95% confluence in six-well plates. Cell layers were scratched using a 20-µL tip to form wound gaps, washed twice with PBS and cultured. The wound healing was photographed at different time points, and each wound was analyzed by measuring the distance migrated by the cells in three different areas.

### Cell invasion assay

A cell invasion assay was performed using BD Matrigel invasion chambers (BD Biosciences) following the manufacturer's protocol. Cells were suspended in serum-free DMEM culture medium at a concentration of 4×10^5^ cells/mL and then added to the upper chamber (4×10^4^ cells/well) with a Matrigel-coated filter. Simultaneously, the lower chambers were filled with 10% FBS as a chemoattractant, and the cells were incubated in the chambers for 48 h. At the end of the experiments, the cells on the upper surface of the membranes were removed using a cotton swab, and the cells on the lower surface were fixed and stained with 0.1% crystal violet. Five visual fields of each insert were randomly chosen and counted under a microscope (IX71, Olympus, Japan). Image-Pro Insight (Version 8.0.21) software was used to count the migrated and non-migrated cells. The mean number of migrating or invading cells was expressed as a percentage relative to the control.

### Apoptosis assay

Cells were harvested and washed in cold PBS. Combined Annexin V and propidium iodide staining was performed using the Annexin V-FITC apoptosis detection kit (BD Biosciences, San Jose, CA) according to the manufacturer's protocol. After the flow cytometric analysis of the cells, apoptosis profiles were obtained with the Cell Quest Pro software.

### Statistical analysis

The data shown are presented as the mean ± SD of at least three independent experiments. The differences were considered statistically significant when p <0.05. A survival curve was estimated using the Kaplan-Meier method in SPSS 13.0, and the resulting curves were compared using the log-rank test.

## Results

### Aberrant expression of miR-181b-5p in human astrocytomas

**Figure 1:**
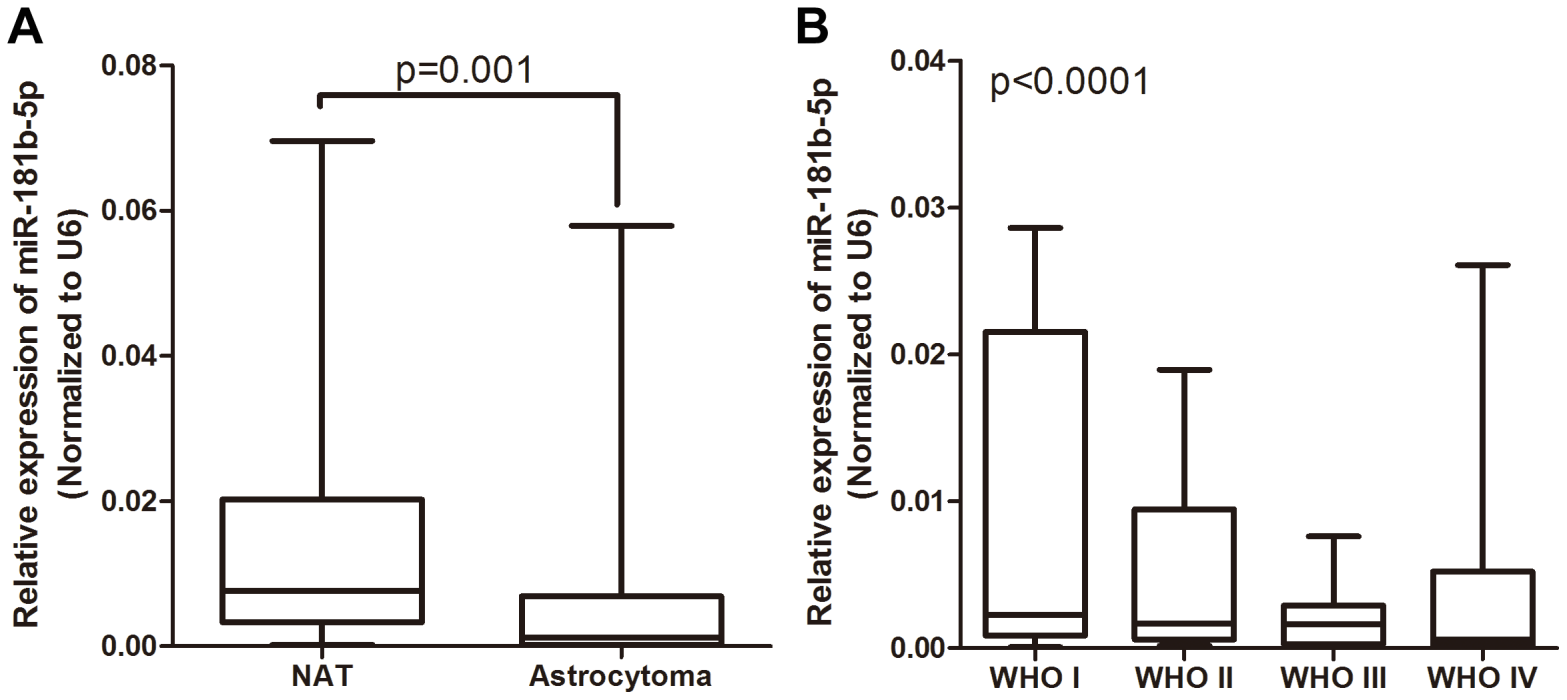
miR-181b-5p is downregulated in astrocytoma tissue samples. The miR-181b-5p expression was normalized to U6. (A) Relative expression of miR-181b-5p in NAT and astrocytoma tissues. (B) Relative expression of miR-181b-5p in different WHO grades of cancer tissues.

To assess the expression of miR-181b-5p in astrocytomas, a qRT-PCR analysis was performed on 25 NAT samples and 90 astrocytoma tissue samples. The results showed that the expression of miR-181b-5p was consistently lower in the astrocytoma tissues compared with the NAT samples (Figure 1A). We then divided the astrocytoma samples into 4 groups according to their tumor grade. As shown in Figure 1B, the expression of miR-181b-5p in the high-grade astrocytomas was significantly lower than in the low-grade astrocytomas. The expression of miR-181b-5p was progressively decreased from the WHO grade I to the WHO grade IV astrocytomas. These data support the notion that low miR-181b-5p expression is closely related to astrocytoma progression and that this miRNA may act as a tumor suppressor in astrocytoma.

### miR-181b-5p overexpression inhibits cell proliferation, migration and invasion and induces apoptosis *in vitro*

**Figure 2:**
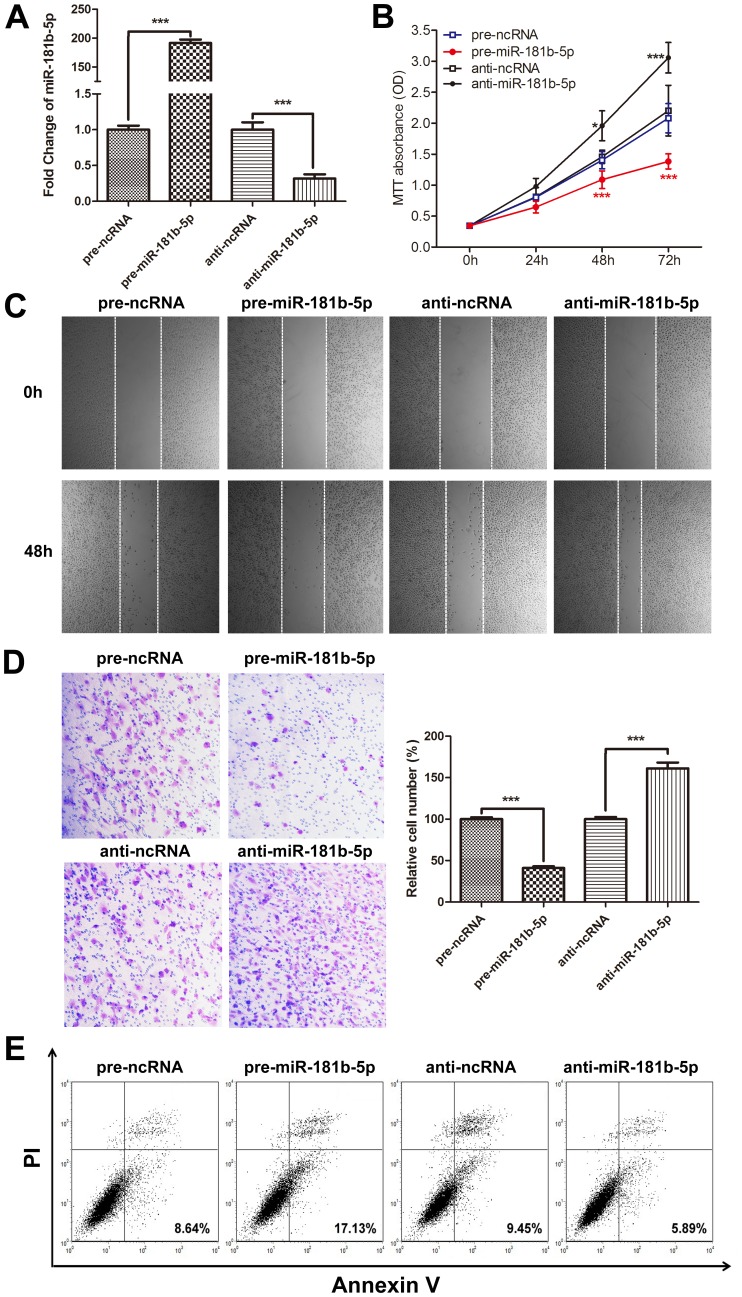
The role of miR-181b-5p in cell proliferation, migration, invasion and apoptosis *in vitro*. (A) Overexpression or knockdown of miR-181b-5p. pre-ncRNA, pre-miR-181b-5p, anti-ncRNA or anti-miR-181b-5p was transfected. The intercellular levels of miR-181b-5p were evaluated by qRT-PCR at 24 h after transfection. For comparison, the expression levels of miR-181b-5p in pre-ncRNA- or anti-ncRNA transfected cells were arbitrarily set at 1. The results are presented as the mean ± SD of three independent experiments (*** p<0.001). (B) The role of miR-181b-5p in cell proliferation. An MTT cell viability assay was performed at 0, 24, 48 and 72 h after the transfection of U251 cells with equal concentrations of pre-ncRNA, pre-miR-181b-5p, anti-ncRNA and anti-miR-181b-5p. For comparison, the expression levels of miR-181b-5p in pre-miR-181b-5p- or anti-miR-181b-5p transfected cells were compared with their respective negative controls (* p<0.05, *** p<0.001). The experiment was repeated three times. (C) Wound-healing assays of U251 cells treated with equal concentrations of pre-ncRNA, pre-miR-181b-5p, anti-ncRNA and anti-miR-181b-5p. The wound gaps were photographed and measured. The images shown are representative images from three independent experiments. (D) Transwell assays of U251 cells treated with equal concentrations of pre-ncRNA, pre-miR-181b-5p, anti-ncRNA and anti-miR-181b-5p. The images shown are representative images from three independent experiments, and a statistical analysis was performed (mean ± SD, *** p<0.001). (E) The role of miR-181b-5p in apoptosis. U251 cells were transfected with equal concentrations of pre-ncRNA, pre-miR-181b-5p, anti-ncRNA and anti-miR-181b-5p. The experiment was repeated three times, and representative data are shown.

To investigate the biological functions of miR-181b-5p in astrocytoma cells, U251 cells were transfected with equal concentrations of pre-ncRNA, pre-miR-181b-5p, anti-ncRNA or anti-miR-181b-5p and analyzed for cell growth. The expression of miR-181b-5p was significantly increased by the introduction of pre-miR-181b-5p, whereas anti-miR-181b-5p abolished the miR-181b-5p levels in U251 cells (Figure 2A). The proliferation assay revealed that the cells that were transiently transfected with pre-miR-181b-5p proliferated at a significantly reduced rate; in contrast, the cells transfected with anti-miR-181b-5p proliferated at a significantly enhanced rate (Figure 2B). The relative cell survival rate of the pre-miR-181b-5p-transfected cells at 72 h was 66.5%, and the relative cell survival rate of the anti-miR-181b-5p-transfected cells at 72 h was 138.7%. To further detect whether miR-181b-5p is associated with the migration ability of astrocytoma, we analyzed the effect of miR-181b-5p expression on the migratory and invasive behavior of U251 cells. The overexpression of miR-181b-5p decreased the migration capacity of astrocytoma cells, whereas the inhibition of miR-181b-5p promoted migration (Figure 2C). Furthermore, the overexpression of miR-181b-5p induced by pre-miR-181b-5p transfection reduced the invasive ability of U251 cells, whereas the knockdown of miR-181b-5p significantly enhanced this ability (Figure 2D). Next, we used Annexin V and PI double-staining FACS analysis to investigate the effects of miR-181b-5p overexpression on astrocytoma cell apoptosis. As shown in Figure 2E, the overexpression of miR-181b-5p induced by transfection with pre-miR-181b-5p resulted in a significant increase in apoptotic cells, whereas the inhibition of miR-181b-5p slightly reduced the number of apoptotic cells. The biological functions of miR-181b-5p on cell proliferation, migration, invasion and apoptosis were also investigated in U87 cells. As shown in Figure S1 in File S1, overexpression of miR-181b-5p significantly inhibited the proliferation (Figure S1A in File S1), migration (Figure S1B in File S1), invasion (Figure S1C in File S1) and promoted cellular apoptosis (Figure S1D in File S1) in U87 cells, while the inhibition of miR-181b-5p had opposite effects. These results showed that miR-181b-5p expression contributes to the regulation of astrocytoma cell proliferation and motility *in vitro*.

### Nova1 Is A Direct Target Of Mir-181b-5p

**Figure 3:**
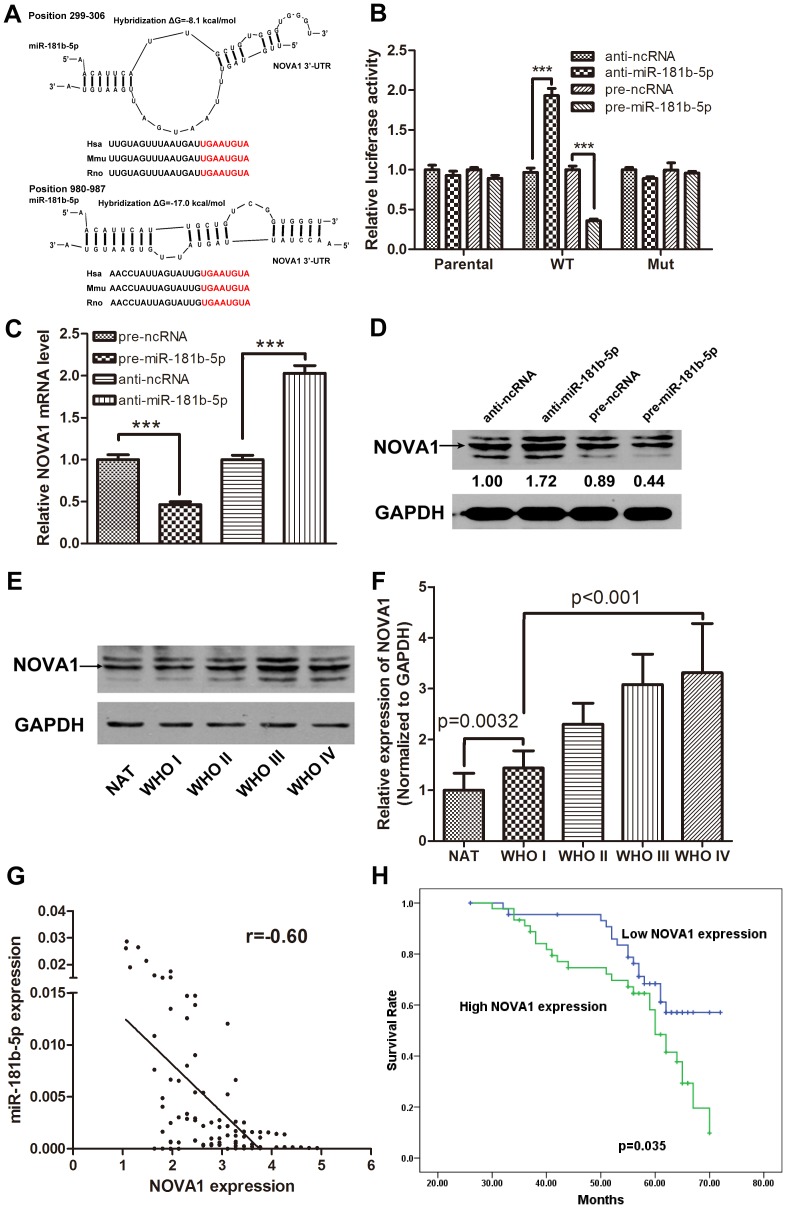
NOVA1 is a direct target gene of miR-181b-5p. (A) A schematic description of the hypothesized duplexes formed by interactions between the NOVA1 3′-UTR binding sites and miR-181b-5p. The predicted free energy of each hybrid is indicated. (B) Direct recognition of the NOVA1 3′-UTR by miR-181b-5p. Firefly luciferase reporters containing either wt or mut NOVA1 3′-UTRs were co-transfected into U251 cells with pre-miR-181b-5p, anti-miR-181b-5p and their corresponding negative controls. The parental luciferase plasmid was also transfected as a control. At 24 h post-transfection, the cells were assayed using luciferase assay kits. The results are presented as the mean ± SD of three independent experiments (*** p<0.001). (C) Quantitative real time-PCR analysis of NOVA1 mRNA expression levels in U251 cells treated with pre-ncRNA, pre-miR-181b-5p, anti-ncRNA and anti-miR-181b-5p. The results shown represent data from three independent experiments (*** p<0.001). (D) Representative western blots showing NOVA1 protein levels in U251 cells treated with pre-ncRNA, pre-miR-181b-5p, anti-ncRNA and anti-miR-181b-5p. (E) Representative western blots showing NOVA1 protein levels in NAT samples and WHO I-IV astrocytomas. (F) Statistical analysis of relative expression of NOVA1 in NAT samples and WHO I-IV astrocytomas. The expression of NOVA1 in NAT samples were arbitrarily set at 1. (G) Spearman's correlation analysis was used to determine the correlation between the expression levels of NOVA1 and miR-181b-5p in human astrocytoma specimens. (H) The relationship between NOVA1 expression and astrocytoma patient survival time.

To fully understand the mechanisms of miR-181b-5p action in astrocytoma, we used three computational algorithms, miRanda, TargetScan and Pictar, to search for potential targets of miR-181b-5p and selected NOVA1 for further analysis. The predicted interaction between miR-181b-5p and its target binding sites within the NOVA1 3′-UTR is illustrated in Figure 3A. To determine whether the negative regulatory effects of miR-181b-5p on NOVA1 expression were mediated through binding to the presumed complementary sites at the 3′-UTR of NOVA1, we fused the entire NOVA1 3′-UTR into a downstream position of a firefly luciferase reporter plasmid. The resulting plasmid was introduced into U251 cells combined with a transfection control plasmid (β-gal) as well as pre-ncRNA, pre-miR-181b-5p, anti-ncRNA or anti-miR-181b-5p. As shown in Figure 3B, miR-181b-5p overexpression significantly decreased the luciferase reporter activity (normalized against β-gal activity) compared with the pre-ncRNA treatment, whereas the inhibition of miR-181b-5p significantly increased the reporter activity. However, transfection with the parental luciferase plasmid (without the NOVA1 3′-UTR) or with the mutant luciferase plasmid (the NOVA1 3′-UTR with mutations in the seed complementary site) did not affect the luciferase reporter activity. We next investigated whether miR-181b-5p could regulate NOVA1 at both the mRNA and protein levels. miR-181b-5p inhibitors or mimics were transfected into cancer cells, and the levels of NOVA1 mRNA and protein were monitored. A qRT-PCR analysis revealed that the inhibition of miR-181b-5p in U251 cells led to increased expression of endogenous NOVA1 mRNA compared with the control; conversely, the enhanced expression of miR-181b-5p decreased the expression of endogenous NOVA1 mRNA compared with the control (Figure 3C). The expression levels of NOVA1 protein were assessed by a western blot analysis at 48 h after transfection. All of the cells transfected with pre-miR-181b-5p exhibited reduced NOVA1 expression relative to the cells transfected with pre-ncRNA, whereas the cells transfected with anti-miR-181b-5p exhibited enhanced NOVA1 expression relative to the cells transfected with anti-ncRNA (Figure 3D). These results strongly demonstrate that NOVA1 is a target gene of miR-181b-5p, which directly recognizes the 3′-UTR of the NOVA1 transcript to downregulate its expression. As NOVA1 is the direct target gene of miR-181b-5p, we asked whether NOVA1 was upregulated in human samples. Figure 3E displays representative western blots of NOVA1 in astrocytomas. The expression of NOVA1 in the astrocytomas was significantly higher than that in the NAT samples, and the expression levels increased as the tumor grade increased. The relative expression changes of NOVA1 in astrocytomas are shown in Figure 3F. To determine whether reduced miR-181b-5p expression correlates with levels of NOVA1 expression in tumor tissues, Spearman's correlation analysis was carried out. The result showed that there was an inverse correlation between the expression levels of NOVA1 and miR-181b-5p (r = −0.60) in human astrocytomas (Figure 3G). In our previous studies, we found that the reduced expression of miR-181b-5p was significantly associated with poor survival outcome, so we asked whether NOVA1 upregulation was correlated with patient survival. As shown in Figure 3H, patients with high NOVA1 expression levels exhibited poorer survival outcomes than patients with low NOVA1 expression levels (p = 0.035). These results further confirm the negative regulation of NOVA1 by miR-181b-5p.

### Knockdown Of Nova1 Inhibits Cellular Proliferation, Migration And Invasion And Induces Apoptosis *In Vitro*

**Figure 4:**
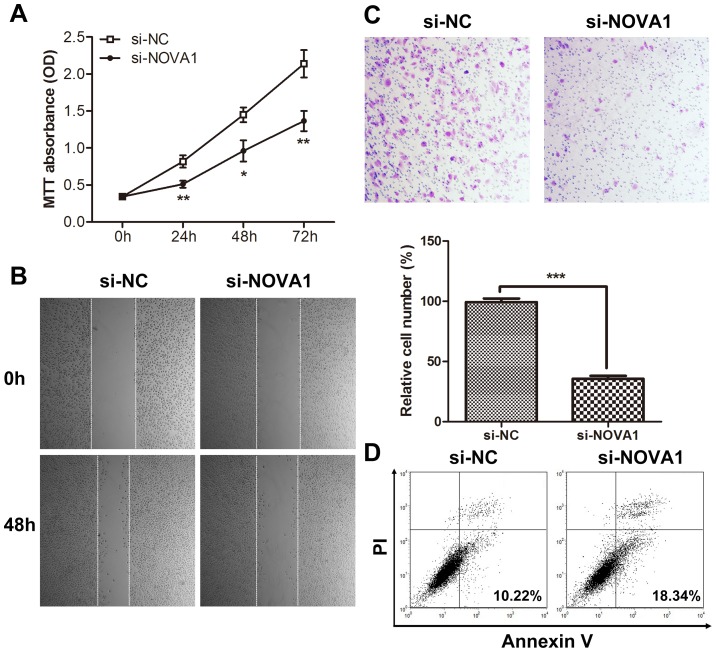
NOVA1 downregulation inhibited cell proliferation, migration and invasion and promoted apoptosis *in vitro*. (A) Downregulation of NOVA1 decreased cell growth (* p<0.05, ** p<0.01). The experiment was repeated three times. (B) Downregulation of NOVA1 decreased cell migration ability. (C) Downregulation of NOVA1 decreased cell invasion ability. The images shown are representative images from three independent experiments, and a statistical analysis was performed (mean ± SD, *** p<0.001). (D) Downregulation of NOVA1 promoted apoptosis.

To determine the effects of NOVA1 on cancer cell proliferation, migration, invasion, and apoptosis, we performed the targeted knockdown of NOVA1 expression using RNAi in U251 cells. A cell proliferation assay showed that NOVA1 knockdown significantly inhibited cell growth (Figure 4A). Consequently, wound-healing and Transwell assays examining migration and invasion were performed. Decreased migrating and invading U251 cells were observed when transfected with si-NOVA1 compared to the controls (Figure 4B-4C). Lastly, an apoptosis assay showed that NOVA1 knockdown promoted apoptosis (Figure 4D). These results were similar in U87 cells, as shown in Figure S2 in File S1.

### Overexpression Of Nova1 Reverses The Inhibitory Effects Of Mir-181b-5p

**Figure 5:**
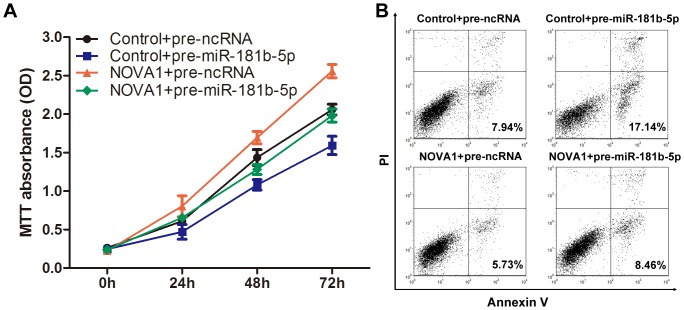
Overexpression of NOVA1 reversed the inhibitory effects of miR-181b-5p. (A) Cell proliferation assay. An MTT cell viability assay was performed at 0, 24, 48 and 72 h after the transfection of U251 cells with control vector + pre-ncRNA, control vector + pre-miR-181b-5p, NOVA1 vector + pre-ncRNA, and NOVA1 vector + pre-miR-181b-5p. The experiment was repeated three times. (B) Cell apoptosis assay. U251 cells were transfected with control vector + pre-ncRNA, control vector + pre-miR-181b-5p, NOVA1 vector + pre-ncRNA, and NOVA1 vector + pre-miR-181b-5p. The experiment was repeated three times, and representative data were shown.

As shown above, overexpression of miR-181b-5p inhibited proliferation, migration, and invasion of astrocytoma cells. We also validated NOVA1 as a direct target of miR-181b-5p. Similar to miR-181b-5p overexpression, we found that reduced expression of NOVA1 significantly inhibited cell proliferation, migration and invasion, similar to those induced by miR-181b-5p. To further elucidate whether the tumor suppressive effect of miR-181b-5p was mediated by repression of NOVA1 in astrocytoma cells, a NOVA1 expression vector which encoded the entire coding sequence without the 3′-UTR was transfected into U251 cancer cells. As shown in Figure 5, ectopic expression of NOVA1 significantly prevented the suppression of proliferation (Figure 5A) and apoptosis (Figure 5B) induced by miR-181b-5p, indicating that NOVA1 was directly responsible for the biological effects induced by miR-181b-5p overexpression.

## Discussion

Accumulating evidence has indicated that the aberrant expression of miRNAs may be a common mechanism involved in the development of various cancers [15]. Regardless, further investigation of cancer-specific miRNAs and their targets is necessary to fully understand their role in the pathogenesis of tumors and may be important for the design of novel therapeutic targets [16].

In our previous study, we established a unique molecular diagnostic signature for astrocytomas that included miR-21-5p, miR-24-3p, miR-30c-5p, miR-106a-5p, miR-124-3p, miR-137 and miR-181b-5p [7]. miR-106a-5p and miR-181b-5p are two of the most significantly downregulated miRNAs in astrocytomas, and their low expression levels are significantly associated with a poor survival outcome; this observation triggered our interest in investigating their function and their target genes during astrocytoma development. In our recent work, we proved that miR-106a-5p inhibits the proliferation and migration of astrocytoma cells and promotes apoptosis by targeting FASTK [10]. However, the potential role of miR-181b-5p as an oncogene or a tumor suppressor in cancer development remains controversial. miR-181b-5p promotes cell proliferation, increases cell migration and invasion and inhibits apoptosis in prostate cancer [17] and gastric cancer by targeting *TIMP3* [18]. miR-181b-5p also enhances drug resistance in breast cancer [19]–[20]. In contrast, this miRNA inhibits cell proliferation and colony formation and induces apoptosis in gastric cancer by targeting *CREB1* [21] and reduces drug resistance in pancreatic cancer by inhibiting *CYLD* and *BCL-2* [22]–[23]. Previous studies have reported that miR-181b-5p functions in gliomas to suppress growth by targeting the *IGF-1R* oncogene [8] and modulates glioma cell sensitivity to temozolomide by targeting *MEK1* [9]. However, it is now well known that miRNAs regulate the expression of multiple target genes and affect a variety of cellular pathways. Here, consistent with our previous studies, we found that miR-181b-5p was downregulated in human astrocytoma tissues. The ectopic expression of miR-181b-5p suppressed cell proliferation, migration and invasion and induced apoptosis *in vitro*. On the basis of a bioinformatic analysis, we further confirmed NOVA1 as a direct target of miR-181b-5p. Therefore, this study may provide new therapeutic strategies for astrocytoma prevention and treatment.

NOVA1 belongs to the Nova family of neuron-specific RNA-binding proteins, which were originally identified as targets in an autoimmune neurologic disease characterized by the failure of motor inhibition. NOVA1 is essential for the proper development of the mammalian motor system and for the survival of motoneurons by controlling the alternative processing of a wide array of mRNAs that are important for synaptic activity [24]. NOVA1 is known as a splicing factor that regulates the inclusion or exclusion of exons depending on the position of NOVA binding relative to splice sites: when NOVA1 binds to the 3′-region of the cassette exon, it promotes the inclusion of this exon [25]. NOVA1 also regulates the alternative splicing of pre-mRNAs encoding the inhibitory neurotransmitter receptor subunits GABA(A)Rgamma2 and GlyRalpha2 by directly binding to intronic elements, resulting in the enhancement of exon inclusion [26]. The antagonistic role of NOVA1 is exemplified in the alternative splicing of D2R (dopamine D2 receptor) pre-mRNA as a pre-mRNA-binding protein [27]. NOVA1 is also an essential regulator of Z^+^_agrin, which induces AChR clusters through interaction with the agrin receptor (Lrp4), leading to phosphorylation of the muscle-specific receptor tyrosine kinase MuSK [28]. Furthermore, NOVA1 might mediate neuronal responsiveness, and its expression might positively correlate with neural repair after ischemia/reperfusion insults in the rat brain [29]. In our study, miR-181b-5p directly targeted NOVA1 expression in astrocytoma cells, playing important roles in disease progression. The overexpression of miR-181b-5p and corresponding reduced expression of NOVA1 decreased the oncogenic potential of cells, as evidenced by decreases in the proliferation rate and cell migration and increased apoptosis damage. The mechanism by which miR-181b-5p decreases the oncogenic potential of cells is most likely through the inhibition of NOVA1. NOVA1 is expressed only in the central nervous system (CNS) in mammals and has been shown to regulate ∼700 alternatively spliced exons by binding to YCAY clusters in target pre-mRNAs [25],[30]. Importantly, most of these targets are genes involved in synaptic function and axon guidance; thus, NOVA1 regulation plays an important role in shaping neuronal wiring and function [31]. The aberrant expression of NOVA1 caused by miR-181b-5p may lead to the inappropriate alternative splicing of oncogenes, which may facilitate astrocytoma pathogenesis. To the best of our knowledge, this study is the first report showing the direct regulation of NOVA1 by miR-181b-5p.

In summary, our data indicate that miR-181b-5p is a tumor suppressor gene in astrocytomas. The overexpression of miR-181b-5p inhibits astrocytoma cell proliferation, migration and invasion and promotes apoptosis. Moreover, we show that NOVA1 is a direct target of miR-181b-5p. Although much remains to be elucidated in terms of the role of miR-181b-5p in the pathogenesis of astrocytomas, miR-181b-5p represents a new potential therapeutic target for the treatment of this cancer.

## Supporting Information

File S1 **Supporting figures.** Figure S1, The role of miR-181b-5p in U87 cell proliferation, migration, invasion and apoptosis *in vitro*. (A) The role of miR-181b-5p in cell proliferation. An MTT cell viability assay was performed at 0, 24, 48 and 72 h after the transfection of U87 cells with equal concentrations of pre-ncRNA, pre-miR-181b-5p, anti-ncRNA and anti-miR-181b-5p. For comparison, the expression levels of miR-181b-5p in pre-miR-181b-5p- or anti-miR-181b-5p transfected cells were compared with their respective negative controls (* p<0.05, *** p<0.001). The experiment was repeated three times. (B) Wound-healing assays of U87 cells treated with equal concentrations of pre-ncRNA, pre-miR-181b-5p, anti-ncRNA and anti-miR-181b-5p. The wound gaps were photographed and measured. The images shown are representative images from three independent experiments. (C) Transwell assays of U87 cells treated with equal concentrations of pre-ncRNA, pre-miR-181b-5p, anti-ncRNA and anti-miR-181b-5p. The images shown are representative images from three independent experiments, and a statistical analysis was performed (mean ± SD; *** p<0.001). (D) The role of miR-181b-5p in apoptosis in U87 cells. U87 cells were transfected with equal concentrations of pre-ncRNA, pre-miR-181b-5p, anti-ncRNA and anti-miR-181b-5p. The experiment was repeated three times, and representative data are shown. Figure S2, NOVA1 downregulation inhibited cell proliferation, migration and invasion and promoted apoptosis in U87 cells. (A) Downregulation of NOVA1 decreased U87 cell growth (** p<0.01, *** p<0.001). The experiment was repeated three times. (B) Downregulation of NOVA1 decreased U87 cell migration ability. (C) Downregulation of NOVA1 decreased U87 cell invasion ability. The images shown are representative images from three independent experiments, and a statistical analysis was performed (mean ± SD; *** p<0.001). (D) Downregulation of NOVA1 promoted apoptosis. (DOC)
