# Correction: MiR-181b-5p Downregulates NOVA1 to Suppress Proliferation, Migration and Invasion and Promote Apoptosis in Astrocytoma

**DOI:** 10.1371/journal.pone.0306667

**Published:** 2024-07-01

**Authors:** Feng Zhi, Qiang Wang, Danni Deng, Naiyuan Shao, Rong Wang, Lian Xue, Suinuan Wang, Xiwei Xia, Yilin Yang

In Fig 4, the images in panels B and C are uploaded incorrectly. Please see the correct Fig 4 here.

**Fig 4:**
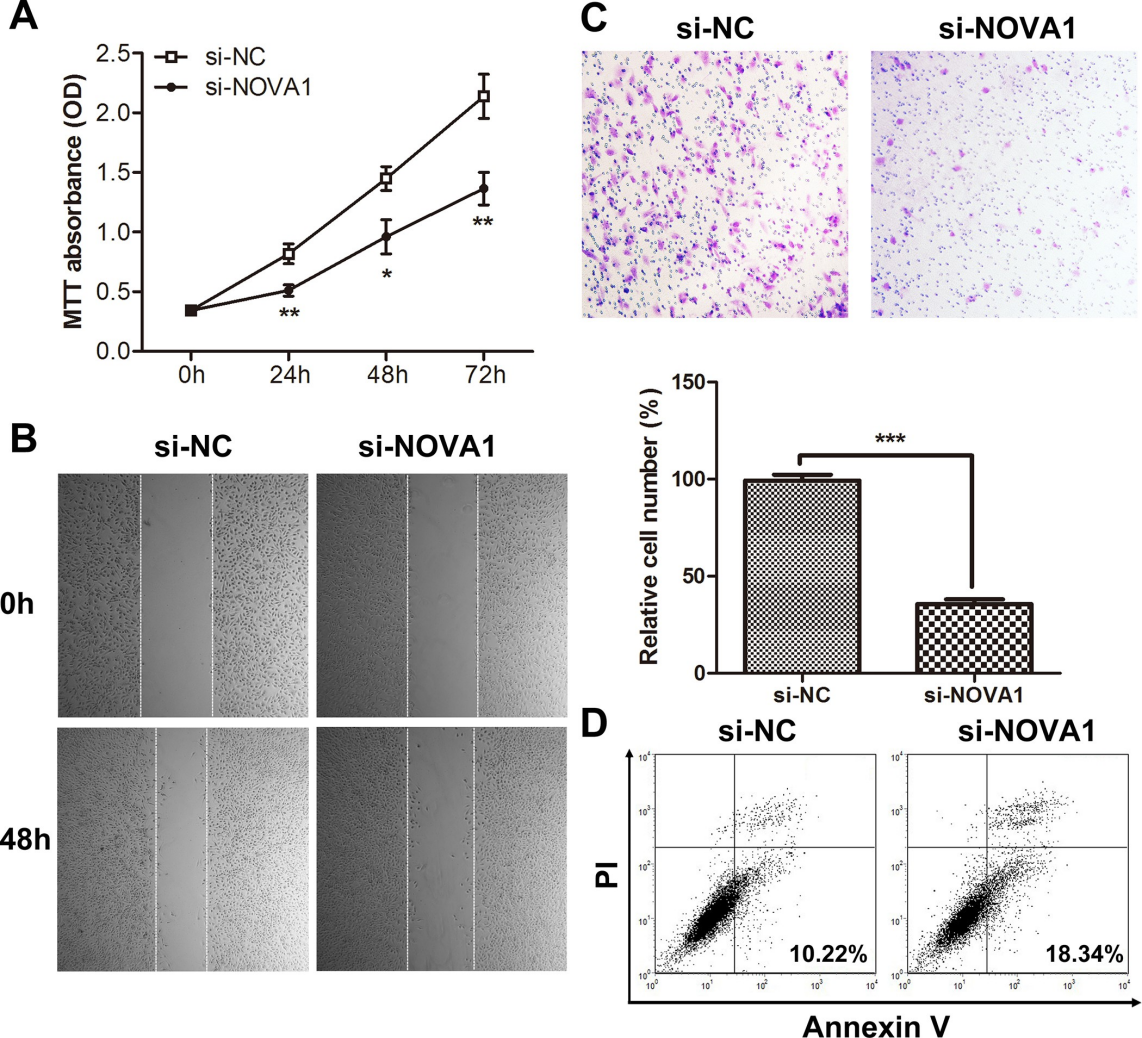
NOVA1 downregulation inhibited cell proliferation, migration and invasion and promoted apoptosis *in vitro*. (A) Downregulation of NOVA1 decreased cell growth (* p<0.05, ** p<0.01). The experiment was repeated three times. (B) Downregulation of NOVA1 decreased cell migration ability. (C) Downregulation of NOVA1 decreased cell invasion ability. The images shown are representative images from three independent experiments, and a statistical analysis was performed (mean ± SD, *** p<0.001). (D) Downregulation of NOVA1 promoted apoptosis.

In File S1, the Figure S1 and S2 are uploaded incorrectly. Please see the correct File S1 here.
