# Supplementary material for: MiR-181b-5p Downregulates NOVA1 to Suppress Proliferation, Migration and Invasion and Promote Apoptosis in Astrocytoma
Source: PLoS One. 2014 Oct 9;9(10):e109124. doi: 10.1371/journal.pone.0109124 (PMC4192361; doi:10.1371/journal.pone.0109124)

**Supporting Information**

**miR-181b-5p downregulates NOVA1 to suppress proliferation, migration and invasion and promote apoptosis in astrocytoma**

Feng Zhi^1,#^, Qiang Wang^2,#^, Danni Deng^1#^, Naiyuan Shao^2^, Rong Wang^1^, Lian Xue^1^, Suinuan Wang^2^, Xiwei Xia^2*^, Yilin Yang^1*^

^1^ Modern Medical Research Center, Third Affiliated Hospital of Soochow University, Changzhou, Jiangsu, China;

^2^ Department of Neurosurgery, Third Affiliated Hospital of Soochow University, Changzhou, Jiangsu, China;

**Key words:** miR-181b-5p, NOVA1, astrocytoma, proliferation, migration, apoptosis

# These authors contributed equally in this work.

*Corresponding authors: Yilin Yang^a^ and Xiwei Xia^b^

^a^E-mail: yilinyang.czfph@gmail.com

^b^E-mail: xia_xiwei@126.com

**Figure S1**. The role of miR-181b-5p in U87 cell proliferation, migration, invasion and apoptosis *in vitro*. (A) The role of miR-181b-5p in cell proliferation. An MTT cell viability assay was performed at 0, 24, 48 and 72 h after the transfection of U87 cells with equal concentrations of pre-ncRNA, pre-miR-181b-5p, anti-ncRNA and anti-miR-181b-5p. For comparison, the expression levels of miR-181b-5p in pre-miR-181b-5p- or anti-miR-181b-5p transfected cells were compared with their respective negative controls (* p < 0.05, *** p < 0.001). The experiment was repeated three times. (B) Wound-healing assays of U87 cells treated with equal concentrations of pre-ncRNA, pre-miR-181b-5p, anti-ncRNA and anti-miR-181b-5p. The wound gaps were photographed and measured. The images shown are representative images from three independent experiments. (C) Transwell assays of U87 cells treated with equal concentrations of pre-ncRNA, pre-miR-181b-5p, anti-ncRNA and anti-miR-181b-5p. The images shown are representative images from three independent experiments, and a statistical analysis was performed (mean ± SD; *** p < 0.001). (D) The role of miR-181b-5p in apoptosis in U87 cells. U87 cells were transfected with equal concentrations of pre-ncRNA, pre-miR-181b-5p, anti-ncRNA and anti-miR-181b-5p. The experiment was repeated three times, and representative data are shown.


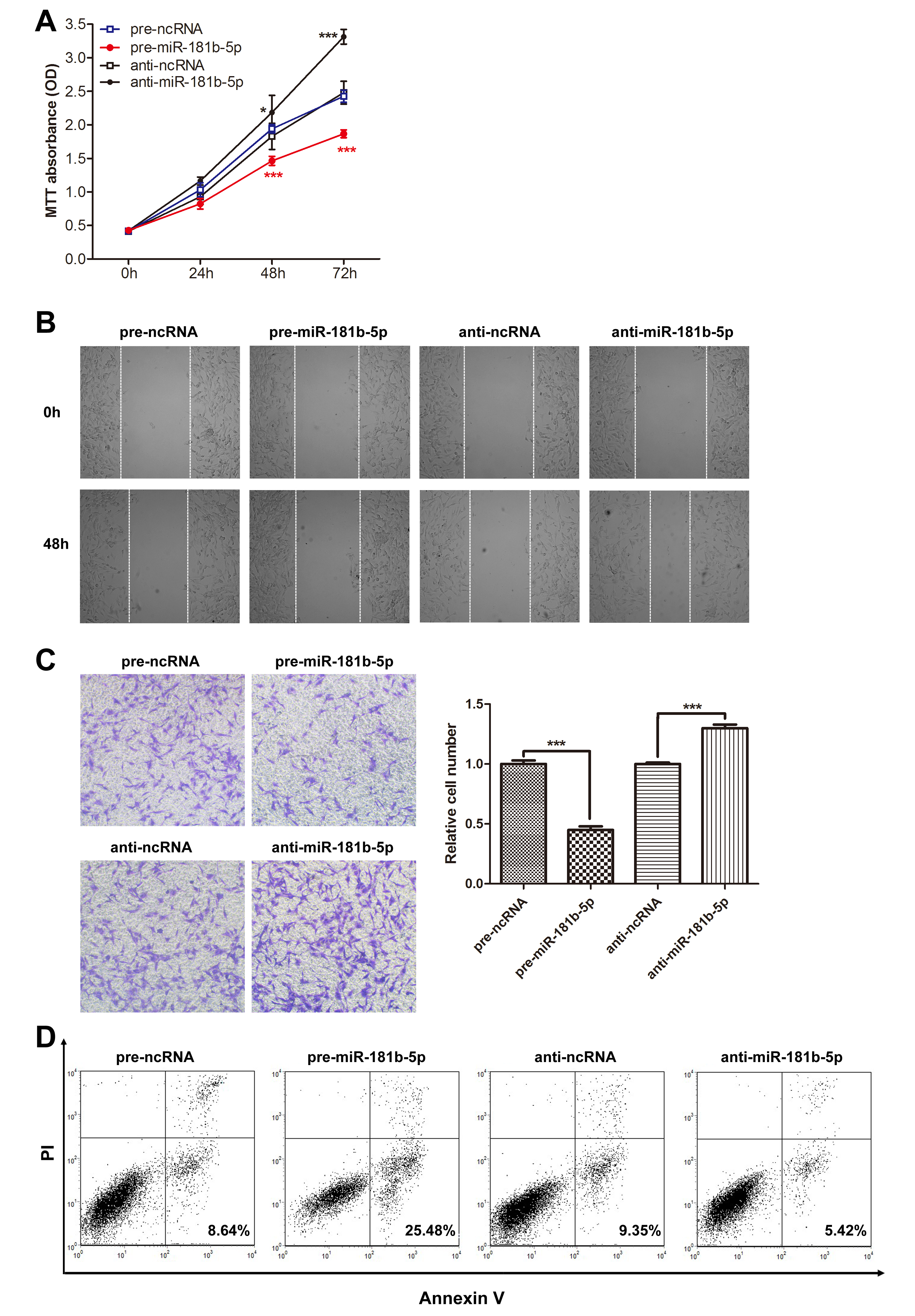


**Figure S2**. NOVA1 downregulation inhibited cell proliferation, migration and invasion and promoted apoptosis in U87 cells. (A) Downregulation of NOVA1 decreased U87 cell growth (** p < 0.01, *** p < 0.001). The experiment was repeated three times. (B) Downregulation of NOVA1 decreased U87 cell migration ability. (C) Downregulation of NOVA1 decreased U87 cell invasion ability. The images shown are representative images from three independent experiments, and a statistical analysis was performed (mean ± SD; *** p < 0.001). (D) Downregulation of NOVA1 promoted apoptosis.


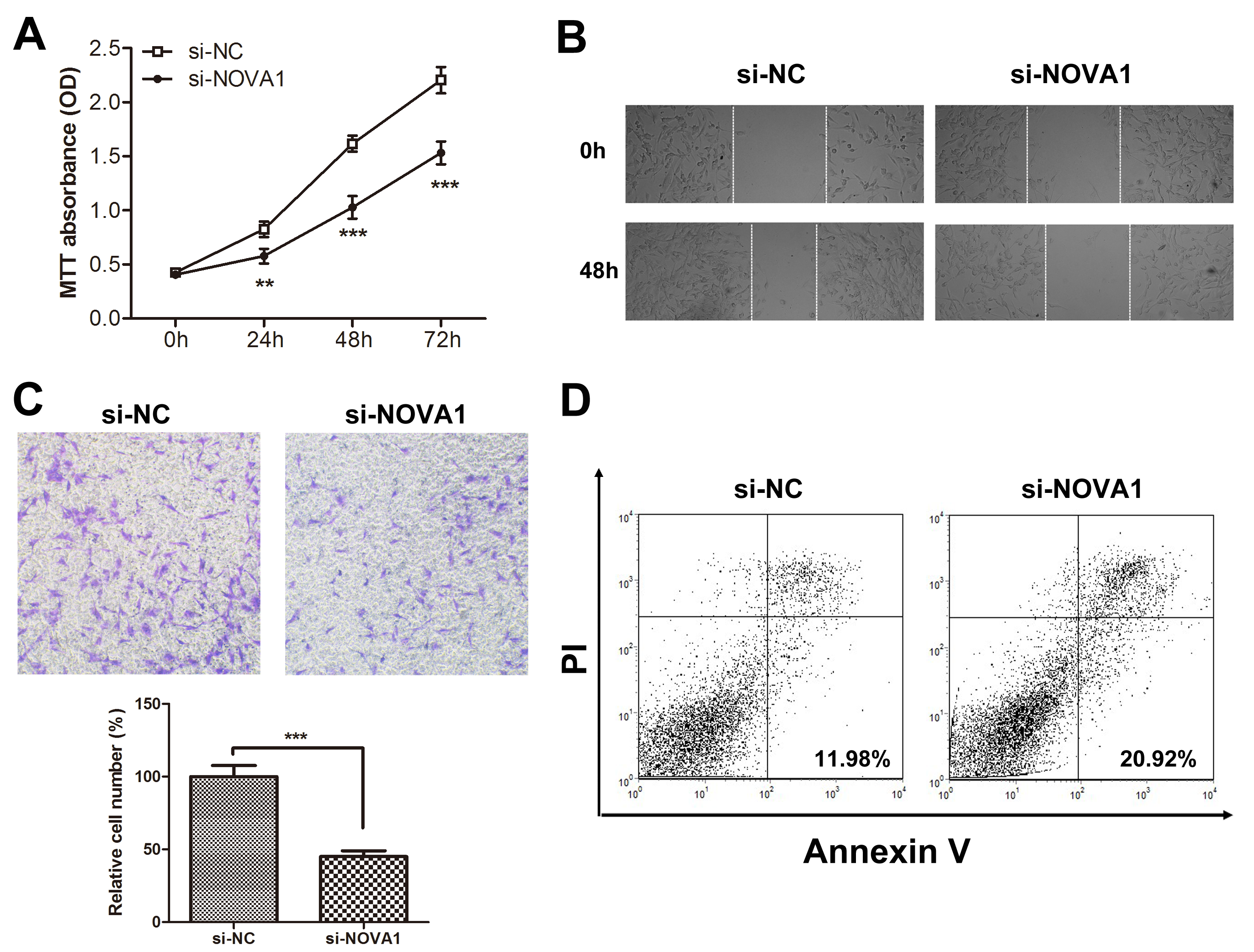

Supplement: File S1 — (DOC) [file pone.0109124.s001.doc]
